# iTRAQ-Based Quantitative Proteomic Analysis Reveals Cold Responsive Proteins Involved in Leaf Senescence in Upland Cotton (*Gossypium hirsutum* L.)

**DOI:** 10.3390/ijms18091984

**Published:** 2017-09-16

**Authors:** Xuewei Zheng, Shuli Fan, Hengling Wei, Chengcheng Tao, Qiang Ma, Qifeng Ma, Siping Zhang, Hongbin Li, Chaoyou Pang, Shuxun Yu

**Affiliations:** 1 State Key Laboratory of Cotton Biology, Institute of Cotton Research of CAAS, Anyang 455000, China; xwzheng0529@163.com (X.Z.); fansl@cricaas.com.cn (S.F.); henglingwei@163.com (H.W.); maqiang5@126.com (Q.M.); maqf@cricaas.com.cn (Q.M.); zhsp5337@163.com (S.Z.); ysx195311@163.com (S.Y.); 2 College of Life Sciences, Key laboratory of Agrobiotechnology, Shihezi University, Shihezi 832003, China; taocc_124@163.com

**Keywords:** premature leaf senescence, iTRAQ, cold-responsive proteins, proteomic, jasmonic acid

## Abstract

Premature leaf senescence occurs in the ultimate phase of the plant, and it occurs through a complex series of actions regulated by stress, hormones and genes. In this study, a proteomic analysis was performed to analyze the factors that could induce premature leaf senescence in two cotton cultivars. We successfully identified 443 differential abundant proteins (DAPs) from 7388 high-confidence proteins at four stages between non-premature senescence (NS) and premature senescence (PS), among which 158 proteins were over-accumulated, 238 proteins were down-accumulated at four stages, and 47 proteins displayed overlapped accumulation. All the DAPs were mapped onto 21 different categories on the basis of a Clusters of Orthologous Groups (COG) analysis, and 9 clusters were based on accumulation. Gene Ontology (GO) enrichment results show that processes related to stress responses, including responses to cold temperatures and responses to hormones, are significantly differentially accumulated. More importantly, the enriched proteins were mapped in The Arabidopsis Information Resource (TAIR), showing that 58 proteins play an active role in abiotic stress, hormone signaling and leaf senescence. Among these proteins, 26 cold-responsive proteins (CRPs) are significantly differentially accumulated. The meteorological data showed that the median temperatures declined at approximately 15 days before the onset of aging, suggesting that a decrease in temperature is tightly linked to an onset of cotton leaf senescence. Because accumulations of H_2_O_2_ and increased jasmonic acid (JA) were detected during PS, we speculate that two pathways associated with JA and H_2_O_2_ are closely related to premature leaf senescence in cotton.

## 1. Introduction

Leaf senescence is an important physiological phenomenon that is defined as an age-dependent progression in the plant’s ultimate phase that leads to death or occurs at the end of the life span [1]. In a senescing leaf, cellular biological changes first occur along with anabolic decline. The synthesis of proteins and RNA decline significantly in leaf cells and the aminopeptidase and endopeptidase activities intensify conspicuously with the degree of leaf senescence. The balance between free radical generation and removal is compromised, resulting in massive accumulation and ultimately exacerbating membrane lipid peroxidation. The integrity of the chloroplasts is in turn largely destroyed relative to that of other organelles during senescence, and thus the chlorophyll content is widely used as a key indicator for judging leaf senescence outcomes [2].

Plants undergo two different evolutionary processes in the natural world. Senescence that occurs early (premature senescence) or late (late maturity) can result from a strong adaptation to the natural habitat, and both play a vital role in determining the annual crop yield production levels [3,4]. Premature leaf senescence in plants is a result of environmental factors and internal interactions, but the key factors that cause this outcome remain unclear. Colder temperatures (20 °C or less) serve as a key predisposing factor, and they contribute to premature leaf senescence. These temperatures can increase the malondialdehyde (MDA) content and decrease the soluble protein and chlorophyll contents, resulting in the inhibition of vegetative growth and decreased net photosynthesis [4,5,6]. They can then lead to accelerated senescence of part of the leaf surface by reducing the lifespan of the photosynthetic machinery and the leaf lifetime carbon gain [7]. Some reports have also indicated that carbon accumulation occurred in the leaves in the form of glucose and fructose under cold conditions, which can in turn serve as a signal to accelerate senescence [4,8,9], suggesting that soluble sugar accumulation was one of the potential triggers of leaf non-sequential senescence [10]. In addition, plant hormones, endogenous factors (abscisic acid (ABA), cytokinin (CK) and ethylene (ET) [1,11,12,13] and other environmental factors (drought [14], salinity [15] and nutrient deficiency [16]) play key roles in senescence regulation.

Cotton (*Gossypium hirsutum* L.) is one of the most economically important crops, and it is widely cultivated for the production of textile fiber materials and cottonseed oil [17]. Premature cotton aging not only leads to a decline in photosynthesis, respiratory function, and proteins, but it also changes the cell-protective enzyme activity, leading to membrane lipid peroxidation [18]. More importantly for cotton plants, premature leaf senescence results in a decline in the cotton yield and quality. Cotton premature leaf senescence has occurred more frequently and with greater severity in China, the USA, and Australia, and it has become a global problem that has restricted cotton production [6,19,20]. Previous studies on leaf senescence have revealed lint yield losses of as much as 20% in the USA [21] and more severe levels in China, with yield declines of up to 50% [22]. Thus, premature leaf senescence constitutes an important constraint on cotton yields and quality, not only in China but also across the globe. Although many efforts have been made, previous studies on leaf senescence have been confined to the physiological, cellular and genomic features of cotton, and there are few proteomic studies on premature senescence.

In an organism, the ultimate realization of the gene function is takes the form of a protein, and changes in the nucleic acid levels cannot accurately reflect changes in proteins. Due to the ability to identify proteins of low abundance or with molecular masses that are too small or large, the isobaric tags for relative and absolute quantitation (iTRAQ) method has been successfully applied in the life sciences to quantify proteins on the basis of peptide labeling and quantification [23]. iTRAQ has also been successfully used for various forms of proteomic analysis (e.g., of cotton anthers [24], fiber [25], and cotton roots and leaves [26]) in previous studies. In the present study, we first used 8-plex iTRAQ to assess the proteome changes and then we identified the differential abundant proteins (DAPs) associated with premature senescence between non-premature senescence (NS) and premature senescence (PS) at four continuous leaf stages. Our results indicated that the onset of cotton premature leaf senescence is tightly linked to a decrease in temperature, and two pathways associated with hydrogen peroxide, jasmonic acid (JA) biosynthesis, and signaling are key factors in shaping cold-induced cotton leaf senescence.

## 2. Results

### 2.1. Leaf Senescence Phenotype Analysis and Physiological Index Determination in Two Cultivars

**Figure 1:**
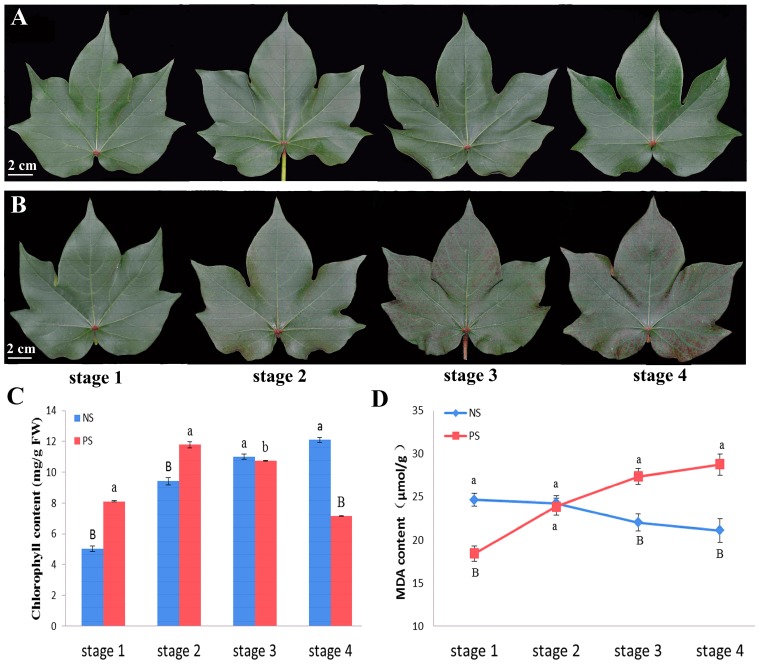
Morphological, chlorophyll and malondialdehyde (MDA) content changes during natural leaf senescence in cotton plants. (**A**) Morphological changes in non-premature senescence (NS) at the four stages; (**B**) Morphological changes in premature senescence (PS) at the four stages; (**C**) Chlorophyll content changes between NS and PS at the four stages; (**D**) MDA content changes between NS and PS at the four stages. The data are the means ± SD (*n* = 3). One-way ANOVA was adopted to perform the statistical analysis. Groups identified by different letters are statistically significant (^a vs. b^ *p* < 0.05; ^a vs. B^ *p* < 0.01).

NS and PS are typical cultivars that were screened in the field to study premature leaf senescence in cotton. During their growth in the field, two cultivars displayed different aging patterns. For the NS in the field, green leaves were maintained (Figure S1A,B) until the actual senescence stage, just like the normal variety. However, more interestingly for PS, a premature senescence variety, the leaves turned reddish brown prior to the actual senescence stage (Figure S1C,D). Therefore, four continuous periods of three days for each stage were used to perform the experiments at 90 days after sowing. As shown in Figure 1A,B, the premature senescence trait appeared at stage 3, at which point reddish brown speckles formed on the PS leaf edges, and then the whole leaves began to turn reddish brown at stage 4. By contrast, the NS leaves remained green throughout the four stages.

The chlorophyll concentrations and MDA content levels were measured as two important indicators of premature senescence (Figure 1C,D). We found that the leaf chlorophyll concentration of the NS increased gradually over time and peaked at stage 4 (Figure 1C). In the PS leaves, the leaf chlorophyll concentration increased from stage 1 to stage 2 and then decreased from stage 3, which was consistent with the phenotypic changes. The NS MDA content also declined slowly from stage 1 to stage 4. However, the levels increased substantially in the PS leaves over all four stages to approximately 1.3 times those of the NS leaves at stage 4, indicating that PS is a typical premature senescence cultivar.

### 2.2. Workflow for the Quantitative Analysis of the Leaf Proteome

**Figure 2:**
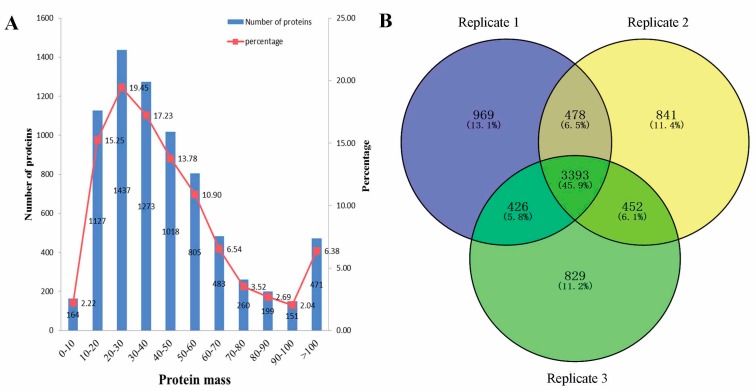
Identification and analysis of proteins that differentially accumulated between NS and PS. (**A**) The proteins were grouped by mass; (**B**) The number of identified proteins generated from a triple isobaric tags for relative and absolute quantitation (iTRAQ) proteomic experiment.

In this study, 8-plex iTRAQ was performed to analyze the proteome differences between the leaves of two cotton cultivars at four stages (Figure 1A,B). Three independent biological replicates were simultaneously performed (Table S1). The data merging after the liquid chromatography-mass spectrometry (LC-MS/MS) analysis generated more than 500,000 mass spectra for each replicate (Table S1). We obtained 49,533, 52,873, and 51,186 unique spectra in each replicate. After using Mascot 2.3.02 to search against the cotton_AD_nr database, 15,025, 15,040, and 14,760 unique peptides were obtained, and ultimately, 5266, 5164, and 5100 proteins were successfully identified in each replicate. We also indirectly show that the data we obtained are relatively high in repeatability and accuracy. The protein mass distribution was also determined. As shown in Figure 2A, the majority of proteins had masses within the 10–60 kDa range, corresponding to a total of 5660 proteins (76.61%).

In a previous study, the genomes of *Gossypium arboretum* (AA) [27], *Gossypium raimondii* (DD) [28], and *Gossypium hirsutum* L. (AADD) were sequenced and assembled. In the present study, 7388 proteins were successfully identified from three replicates (Table S1), representing 17.88%, 18.03% and 9.61% protein-coding loci that were annotated in *Gossypium arboretum*, *Gossypium raimondii* and *Gossypium hirsutum* L., respectively. Therefore, the proteins identified here were representative of cotton leaves. Among these proteins, 3393 (45.9%) were identified in three replicates; 478 (6.5%), 452 (6.1%), and 426 (5.8%) proteins were identifiable in two replicates; and 969 (13.1%), 841 (11.4%), and 829 (11.2%) proteins were found to be specific to each replicate (Figure 2B). We designated 4749 proteins that were detected in at least two replicates as identified proteins (Figure 2B).

### 2.3. Analysis of Cotton Premature Senescence Leaf Differential Abundant Proteins (DAPs)

**Figure 3:**
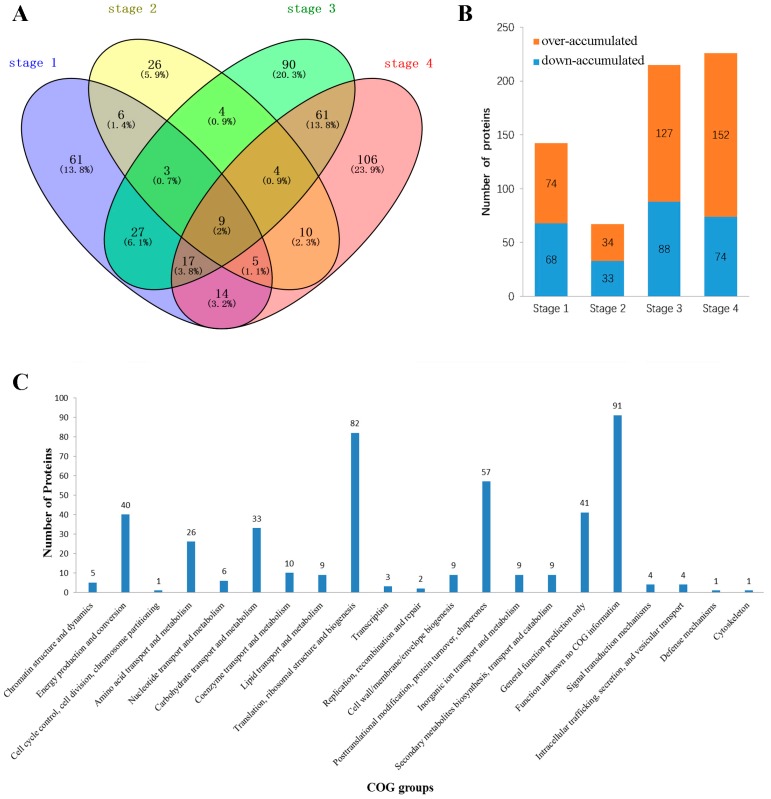
Identification and analysis of differential abundant proteins (DAPs) between NS and PS. (**A**) A DAP distribution Venn diagram between NS and PS; (**B**) Over- and down-accumulated DAPs between NS and PS at the four stages; (**C**) Functional classification of DAPs from leaf proteomes between NS and PS, which were listed as follows: B: chromatin structure and dynamics; C: energy production and conversion; D: cell cycle control, cell division, chromosome partitioning; E: amino acid transport and metabolism; F: nucleotide transport and metabolism; G: carbohydrate transport and metabolism; H: coenzyme transport and metabolism; I: lipid transport and metabolism; J: translation, ribosomal structure and biogenesis; K: transcription; L: replication, recombination and repair; M: cell wall, membrane, envelope biogenesis; O: posttranslational modification, protein turnover, chaperones; P: Inorganic ion transport and metabolism; Q: secondary metabolites biosynthesis, transport and catabolism; R: general function prediction only; S: function unknown no COG information; T: signal transduction mechanisms; U: intracellular trafficking, secretion, and vesicular transport; V: defense mechanisms; Z: cytoskeleton; COG: Clusters of Orthologous Groups.

Here, over- or down-accumulated proteins were determined using the PS/NS ratio, and 443 DAPs were identified at four stages (Figure 3A and Tables S2 and S3) between NS and PS. As shown in Figure 3B, 142 DAPs, 68, 215, and 226 DAPs were significantly accumulated from stage 1 to stage 4. The protein abundance of 9 proteins changed over the four stages that represented 2% of the DAPs (Figure 3A), and 29 and 122 proteins were shared over three and two stages, respectively, suggesting that the emergence of the senescence phenomena was the result of interactions between multiple proteins at various times. Although the number of down-accumulated proteins was greater than the number of over-accumulated proteins over all four stages, the over-accumulated proteins achieved a maximum value of 88 DAPs at stage 3 (Figure 3B). Just as the senescent phenotype appears at stage 3, the down-accumulated proteins showed a significant increase to 152 at stage 4. The number of DAPs increased from stage 1 to 4, indicating that the DAP accumulation showed more coordinated temporal changes during leaf senescence.

The rational classification of proteins is critical in functional annotation and evolutionary studies [29]. A functional prediction was performed to develop a better overall understanding of DAPs in the present study. In total, 443 proteins were successfully mapped to 21 different Clusters of Orthologous Groups (COG) categories (Figure 3C), and function J (translation, ribosomal structure and biogenesis), which was the most frequently detected category, included 82 (18.5%) DAPs. The remaining DAPs are primarily categorized as C (energy production and conversion) with 40 (9.0%) DAPs; E (amino acid transport and metabolism) with 31 (5.9%) DAPs; G (carbohydrate transport and metabolism) with 33 (7.4%) DAPs; O (posttranslational modification, protein turnover, and chaperones) with 57 (12.9%) DAPs; and R (general function prediction only) with 41 (9.3%) DAPs. We found that 91 DAPs did not have corresponding COG information and thus require further study to elucidate their functions during leaf senescence.

### 2.4. GO Enrichment and Cluster Analysis of DAPs

**Figure 4:**
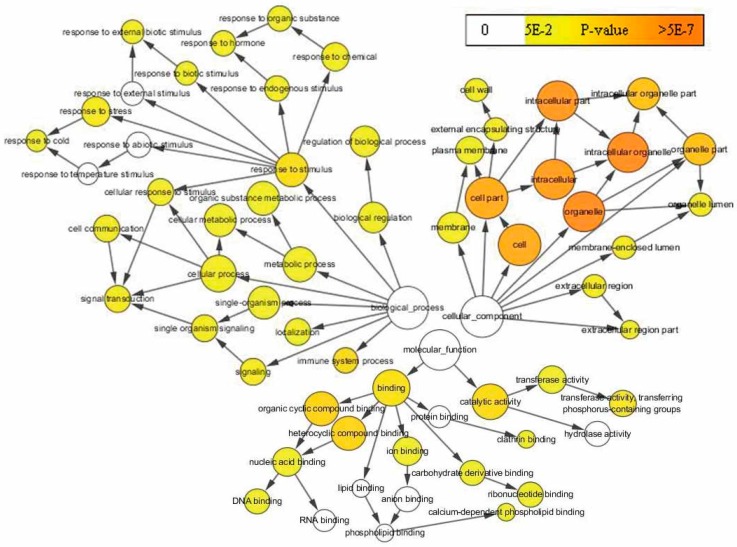
Gene Ontology (GO) enrichment analysis for DAPs during leaf senescence. The network graphs show a BiNGO visualization of the over-represented GO terms for the combined clusters of DAPs during leaf senescence. Colored nodes represent GO terms that are significantly over-represented; the colors are shaded according to the significance levels as shown in the color bar, and the arrow indicates the next biological process is a subset of the former.

In the previous section, the quantification and functional prediction of DAPs at four continuous stages was discussed. Here, a GO enrichment analysis was performed and the result shows that the proteins involved in various biological processes are differentially accumulated during leaf senescence (Figure 4 and Table S4). This finding showed that DAPs were significantly enriched for proteins linked to biological processes that were primarily focused on the response to stimulus, metabolic process, single organism process, biological regulation, and others. Interestingly, processes involved in responses to biotic or abiotic stress, including those involved in responses to stress, immune system processes and signal transduction and others, were enriched at an early stage. It is noteworthy that processes responding to cold or hormones also enriched the accumulated proteins, and these were considered to be a widespread focus. Within the cellular components, the DAPs were primarily located in the organelles, the membranes and the cells to perform their various functions. Finally, within the molecular function, proteins were accumulated in binding and catalytic activity (Figure 4).

**Figure 5:**
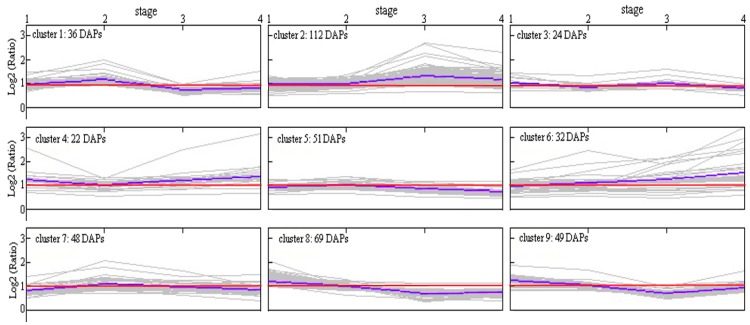
Cluster analysis of DAPs between NS and PS at the four stages. The purple line denotes the mean of the Log2 (ratio) for every cluster. Proteins below the red line are defined as over-accumulated DAPs in the PS.

In addition, the *k*-mean value was used to perform a cluster analysis, and all the DAPs were divided into nine groups as shown in Figure 5. Nine clusters were primarily classified into two categories according to their accumulation profiles. Type I contains 123 DAPs (clusters 3, 5, and 7) that were mostly over-accumulated at four stages for PS, and Type II contains 134 DAPs (clusters 2 and 4) that were mostly down-accumulated at four stages for PS. The remaining clusters displayed a more complex profile, for example, the DAPs in clusters 8 and 9 showed an initial over-accumulation followed by down-accumulation for PS (Figure 5). The GO enrichment analysis showed that over-accumulated DAPs were enriched for proteins linked to biological processes that were primarily focused on signaling, the immune system process, response to stimulus, and metabolic process. Within the cellular components, over-accumulated proteins were primarily located in organelles. Regarding the molecular function, the proteins were primarily focused on catalytic activity (Figure S2). However, the down-accumulated DAPs displayed a different picture. There was enrichment for localization, establishment of localization, the immune system process, the response to stimulus, and biological regulation in the biological process. Within the cellular components, the proteins were primarily located in organelles, cells, and cell parts. The proteins linked to molecular functions were primarily focused on catalytic activity and binding (Figure S2).

### 2.5. Analysis of Gene Expression Levels Associated with DAPs

**Figure 6:**
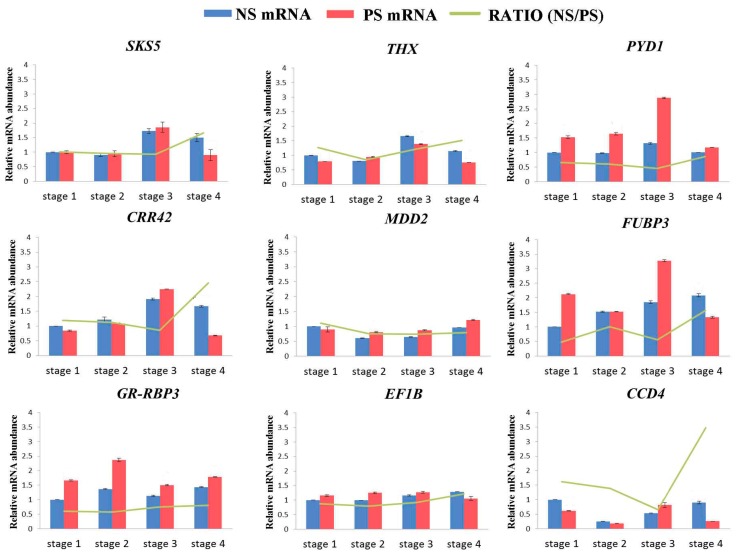
The relative mRNA abundance of genes related to leaf senescence through qPCR between NS and PS. GhActin and NS stage 1 were chosen as the endogenous control and reference sample, respectively. The green line denotes the relative transcript level ratio of NS divided by PS for the corresponding stage. The data are the means ± SD (*n* = 3). *SKS5*: *SKU5 similar 5*; *THX*: thioredoxin X; *PYD1: pyrimidine 1*; *CRR42*: *chlororespiratory reduction 42*; *MDD2*: *mevalonate 5*-*diphosphate decarboxylase 2*; *FUBP3*: *far upstream element*-*binding protein 3*; *GR-RBP3*: *glycine*-*rich RNA*-*binding protein* 3; *EF1B*: *elongation factor EF1B*; *CCD4*: *carotenoid cleavage dioxygenase 4*.

To verify whether the differences in protein abundances were reflected at the transcriptional level and to validate the proteomic analysis [24], qPCR and the 2^−ΔΔ*C*t^ method were used to analyze the level of gene expression associated with DAPs at four continuous stages between NS and PS, and *GhActin* and NS stage 1 were designated the endogenous control and reference sample, respectively. One gene was randomly selected from each cluster, and their expression levels are shown in Figure 6. Only one of the nine candidate genes, *carotenoid cleavage dioxygenase 4* (*CCD4*), followed a similar trend when compared to the results of the cluster analysis (Table S5 and Figure 6, RATIO). Although pyrimidine 1 (PYD1) and glycine-rich RNA-binding protein 3 (GR-RBP3) were over-accumulated in the PS at the protein, the differences in the two expression profiles were significant at more than two stages. The mRNA abundance levels of four genes showed consistent accumulation patterns with those of the proteins at three of the four stages, and they were significantly over-accumulated in NS at stage 1, 3, and 4 (*thioredoxin X*, *THX*) and at stage 1, 2, and 4 (*chlororespiratory reduction 42*, *CRR42*). They were down-accumulated in NS at stage 2, 3, and 4 (*mevalonate* 5-*diphosphate decarboxylase 2*, *MDD2*) or were over-accumulated in PS at stage 1 and over-accumulated in NS at stage 2 and 4 (*far upstream element-binding protein 3*, *FUBP3*), while the remaining stages showed the opposite trend. Furthermore, two genes, *SKU5 similar 5* (*SKS5*) and *translation elongation factor EF1B* (*EF1B*), exhibited different expression patterns than those of the above genes, with the expression profiles that were the same at stage 3, conflicting at the remaining three stages. All of these results indicate poor consistency between the protein and mRNA expression patterns, which is in accordance with similar reports from previous studies [24,30].

### 2.6. DAPs of Arabidopsis Homologs Primarily Associated with Environmental Stresses or Accumulated Plant Hormone Enrichment

Premature senescence processes intrinsically linked to environmental stresses (such as chilling [6,31] and salinity [15,32]), plant hormones [11,12,13], and Senescence Associated Genes (SAGs) [33,34] served as positive regulators that led to aging. Based on the GO annotation in The Arabidopsis Information Resource (TAIR), we found fifty-eight *Arabidopsis* homologous proteins that were primarily divided into 21 categories (Table S6 [35,36,37,38,39,40,41,42]).

Twenty-six cold-responsive proteins (CRPs) of *Arabidopsis* homologs (AT3G12490, AT2G27710, etc.) were significantly accumulated in our data, with 19 CRPs of *Arabidopsis* homologs being over-accumulated and 7 CRPs of *Arabidopsis* homologs being down-accumulated. Regarding the salinity, which was the second-largest environmental factor included in our data, 18 proteins with *Arabidopsis* homologs (AT1G11910, AT5G35080, etc.) were found to have an active role in the plant’s response. Among them, 8 proteins were significantly over-accumulated and 6 proteins were significantly down-accumulated at different stages. In addition to the above factors, many other proteins that respond to heat played a potential role during leaf senescence. Taken together, these results suggest that environmental stress serves as a regulator in promoting premature leaf senescence. In particular, we deduce that cold temperatures may be the most significant environmental factor that affects leaf senescence.

Simultaneously, *Arabidopsis* homolog proteins that responded to plant hormones, including JA (AT2G39730), ABA (AT4G25000, AT4G38970, etc.) and CK (AT5G20630, AT3G14420, and AT3G14420), were also enriched, indicating that plant hormones may be one of the factors that promote or delay senescence. Moreover, the *Arabidopsis* homolog proteins significantly and differentially accumulated for the regulation of programmed cell death (PCD) and aging (AT5G60360, AT5G60360, and AT4G30920), as shown in Table S6, and they may be direct mediators of premature leaf senescence. More importantly, the functions of 12 proteins were verified on the basis of relevant references, which are listed in Table S6.

### 2.7. Temperature Changes Were Linked to the Onset of Premature Leaf Senescence

To ascertain whether temperature changes are correlated with the causes of premature leaf senescence, we consulted the meteorological data for 2013 to 2016. These data included daily maximum, minimum and medial temperatures, monthly rainfall levels, and daily rainfall levels (Table S7).

**Figure 7:**
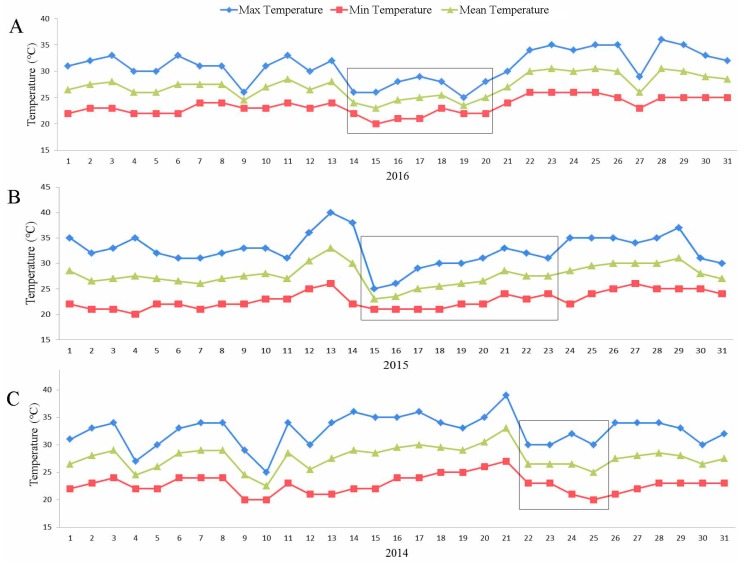

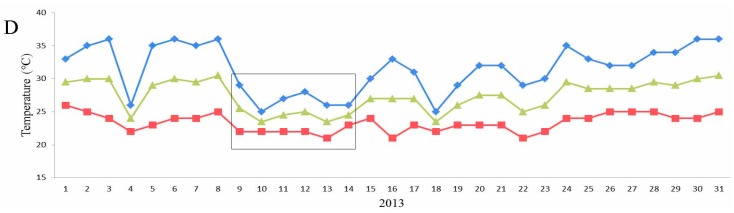
Changes in field temperatures during cotton growth in July from 2013 to 2016. Significant changes in temperature are marked with rectangular boxes. The data were drawn from the Anyang Meteorological Administration and Institute of Cotton Research of the Chinese Academy of Agricultural Sciences (CAAS).

As a common phenomenon, we found a sharp medial temperature decline period in July of each year (Figure 7A–D). This pattern remained stable for a period of time (more than four days). As shown in Table S8, the amplitudes of the temperature decline were 4, 7, 6.5, and 4.5 °C for each year from 2013 to 2016, respectively. It was also interesting to find that the number of days between a sharp decline in temperature and the time of premature leaf senescence onset was approximately 15 (Table S8). We thus conclude that the onset of cotton premature aging is closely related to the decrease in the median temperature, suggesting that the decrease in temperature is a factor that results in premature leaf senescence in addition to other environmental factors. Many reports have shown that drought is another critical factor that promotes premature senescence [43,44], but it was not included in this study. The rainfall levels were found to increase significantly from May to July of each year (Table S7). To confirm the specific rainfall levels for July, the rainfall levels for each day of the month were studied. Significant precipitation events occurred during periods of temperature decline, during which the rainfall levels were precisely correlated with the temperature change time-points. These results show that the water levels were sufficient for cotton growth, thus indicating that drought may not be the factor that promotes premature senescence in this study.

Taken together, the evidence shows that decreases in the temperature served as a regulator of premature leaf senescence, but leaf senescence may be not affected by drought in this study.

### 2.8. Hydrogen Peroxide and JA Levels Were Significantly Increased during Premature Leaf Senescence

**Figure 8:**
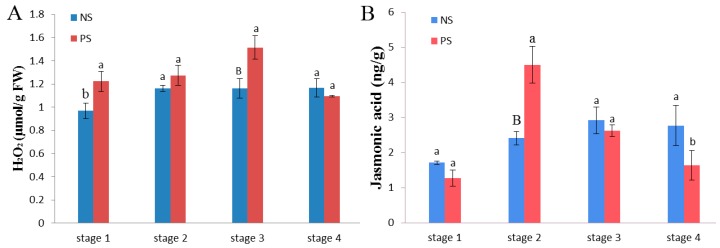
Concentration detection results for two cultivars at the four stages during leaf senescence. (**A**) Concentrations of H_2_O_2_ in NS and PS; (**B**) Concentrations of jasmonate in NS and PS. The data are the means ± SD (*n* = 3). A one-way ANOVA was adopted to perform the statistical analysis. Groups identified by different letters are statistically significant (^a vs. b^ *p* < 0.05; ^a vs. B^ *p* < 0.01).

Catalase 2 was found to be differentially accumulated at stage 3, glycolate oxidase 1 (GOX1) was differentially accumulated at stage 1, and proteins responding to JA were also over-accumulated at later stages (Table S6). Therefore, the concentrations of H_2_O_2_ and JA were measured. As shown in Figure 8, the concentration of H_2_O_2_ continuously increased from stage 1 to stage 3 in PS up to a peak value. The concentration then decreased at stage 4. However, in the NS, the concentration essentially remained stable over the four stages, exhibiting no significant changes. Simultaneously, the JA concentration increased from stage 1 to 2 and then sharply declined from stage 3 to 4 in PS. This finding reveals an obvious difference in comparison to the NS at stage 2, when it reached its maximum. However, the levels rose slightly from stage 1 to 3 in the NS.

### 2.9. SAG2 Was Up-Regulated and Expressed under JA Treatment

**Figure 9:**
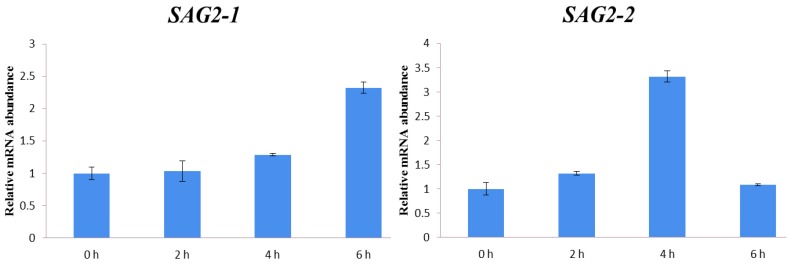
The relative mRNA abundance of *SAG2* in PS under the treatment of JA.

Two cotton genes were confirmed to be *Arabidopsis* homologs (SAGs) according to the Basic Local Alignment Search Tool (BLASTX) result in TAIR; therefore, the primers (Table S9) (CotAD_63566 and CotAD_06563) were designed to investigate the transcriptome level under JA treatment using qRT-PCR. The report showed that SAG may be up-regulated under the plant hormone treatment [45]. Thus, two *Arabidopsis* homologs (Table S6) of *SAG 2* (AT5G60360), which are numbered as *SAG2-1* (CotAD_63566) and *SAG2-2* (CotAD_06563), were obtained here. As shown in Figure 9, their expression levels increased under the JA treatment. The expression level of *SAG2-1* rose from 0 to 6 h all the way; however, for *SAG2-2*, it rose from 0 to 4 h and then declined from 6 h, suggesting that both of them were regulated by JA.

## 3. Discussion

### 3.1. Decrease in Temperature Is One of the Factors Involved in the Premature Leaf Senescence of Cotton

In essence, leaf senescence is a form of PCD that progresses at a much slower rate than, for example, the hypersensitive response induced by pathogens [46]. In this study, many proteins were found to be significantly and differentially accumulated at four stages in relation to cold stress. As the most significant forms of abiotic stress regulating plant developmental stages, environmental factors may play a positive role in leaf senescence. In the present study, 26 CRPs were clearly enriched and accumulated. Cold temperatures have been confirmed to cause an accumulation of sucrose, which induces leaf senescence and a loss of chlorophyll [31], or they have been considered a key predisposing factor that results in cotton leaf senescence [4]. We thus infer that CRPs significantly over-accumulated in these cases, leading to a direct relationship with the temperature decline, thus suggesting that PS is a temperature-sensitive cultivar. Report has also shown that cotton chilling tolerance can be enhanced by mild chilling stress (17 °C for 24 h), indicating cotton might have cold acclimation capacity [47]. Meteorological data were analyzed, and we found a prevalent phenomenon in which declining temperatures occurred approximately 15 days before the onset of premature senescence, suggesting cotton may be acclimated by significant temperature decline for certain periods. Therefore, premature senescence (or non-premature senescence) might be a part of survival strategies under cold temperature. We thus conclude that decline in temperatures may be a factor in inducing cotton leaf senescence.

### 3.2. Hydrogen Peroxide, JA Biosynthesis and Signaling Pathways Are Crucial for Premature Leaf Senescence

Higher hydrogen peroxide additionally contributes to premature senescence in plant leaves [48]. The concentrations of H_2_O_2_ increased from stage 1 to 3, potentially in response to the over-accumulation of GOX1 at stage 1 (Table S6) [49]. The generation of reactive oxygen species (ROS) damaged the photosystem II reaction center and membrane lipids, ultimately leading to plant death [50,51]. Therefore, we surmised that the H_2_O_2_ levels that significantly increased from stage 1 to 3 in PS may be a promoter to induce cell death [52,53]. However, catalase 2 and 2-Cys peroxiredoxin B (2CPB) were also over-accumulated in response to the accumulation of H_2_O_2_ and may be associated with the scavenging of reactive oxygen species (ROS) [54,55], indicating that the H_2_O_2_ signaling pathway plays a role in regulating premature senescence in cotton leaves.

Simultaneously, environmental stress was found to involve various hormones, such as ABA and JA. Although ABA has been shown to promote leaf senescence in several studies in various plants [13,56], we did not find significantly over-accumulated ABA signals in our data. As an emerging positive regulator, JA was shown to be a mediator of the timing of leaf senescence [57,58] as the primary cause of over-accumulated proteins associated with JA from stage 3 to 4, and we thus infer that the JA signaling pathway plays a role in the regulation of premature cotton senescence. According to our data, as a representative hormone associated with senescence, JA significantly increased in PS compared with NS, indicating that the JA biosynthesis pathway is one of the factors involved in premature cotton senescence. Furthermore, a previous study showed that low temperatures can elevate the levels of JA [31,59], indicating that it may also be closely related to cold temperatures in this study. Therefore, JA may be a hormonal trigger while simultaneously accelerating leaf senescence.

In addition, the up-regulation of genes related to environmental stress and hormone signaling was observed during leaf senescence. *SAG29* was found to promote leaf senescence that was induced by salinity, cold temperatures, or ABA [45]. *SAG12*, *SAG29*, *SAG21*, and *SAG113* were differentially expressed in senescent leaves [55] and clearly delayed leaf senescence in a *sag113* knockout mutant line [60]. *SAG2* is also known to be differentially expressed during plant senescence [61]. SAG2 was down-accumulated during stage 1 but over-accumulated at stage 3, and thus we demonstrate that *SAG2* is induced by JA signals and is expressed to mediate leaf senescence during later cotton leaf stages. In summary, we conclude that the two pathways relating to hydrogen peroxide and JA biosynthesis and signaling are closely related to temperature decrease-induced premature leaf senescence in cotton. Many cellular metabolic processes were significantly enriched according to the GO enrichment analysis, such as the cellular macromolecule metabolic process, organic substance biosynthetic or metabolic process, glycine decarboxylation via glycine cleavage system, and others. It was these complex metabolic processes that ultimately led to leaf senescence in cotton.

## 4. Materials and Methods

### 4.1. Plant Growth and Leaf Collection

Two cotton (*G. hirsutum* L.) cultivars, Pinbi 12 (non-premature senescence, NS) and CCRI58 (premature senescence, PS), were collected during this study. Both were grown in a normal agronomic field at the Institute of Cotton Research at the Chinese Academy of Agricultural Sciences (CAAS) in Anyang (E 114°48′, N 36°06′), China, from April to October. The two cultivars are allotetraploid cottons (*G. hirsutum* L.) that share the same genetic background. Three replicates were prepared for each genotype in the field.

The day when the first piece of cotton true leaves had fully expanded was designated ‘day zero’. From the thirtieth day, we picked cotton leaves every three days until the emergence of the PS senescence phenomena. Four continuous periods were selected to perform experiments on each genotype, which~were labeled stage 1 (the sample was taken on 24 July 2016), stage 2 (the sample was taken on 27 July 2016), stage 3 (the sample was taken in 30 July 2016), and stage 4 (the sample was taken in 2 August 2016). Four stages were defined according to the onset time of premature leaf senescence (stage 3) here. Simultaneously, four-leaf cotton seedlings of PS were treated in triplicate using 150 μM JA, and then the leaves (at 0, 2, 4, and 6 h) were stored at −80 °C until RNA isolation was performed. All the samples were immediately frozen in liquid nitrogen and stored at −80 °C until protein and RNA extractions were performed [24].

### 4.2. Protein Extraction, Digestion, and iTRAQ Labeling

The leaf proteins were extracted using a modified phenol method as previously described [62,63]. Three biological replicates were simultaneously performed at each stage. The supernatants were stored at −80 °C until they were needed. All the proteins in the supernatant were quantified using the Bradford method [24].

The protein (200 μg) from each replicate was diluted in 200 μL of buffer (UA; 8 M urea and 150 mM Tris-HCl, pH 8.0). After the addition of 5 μL of DL-Dithiothreitol, (DTT, 1 M) for 1 h at 37 °C, 20 μL of Iodoacetamide (IAA, 1 M) was added [25]. The samples were incubated in the dark for another hour. All the samples were then transferred into a 10 kDa ultrafiltration tube for centrifugation at 12,000 rpm (13,780× *g*) for 20 min. Each filter was rinsed twice with 100 μL of UA buffer, and 100 μL of dissolution buffer (triethylammonium bicarbonate, 50 mM, pH 8.5) was added to the filters, followed by centrifugation for 20 min. This step was repeated three times, and 100 μL of trypsin buffer (100 ng/μL) at a ratio of 1:50 (*w*/*w*) was then added to each filter. All the samples were incubated for 18 h at 37 °C and then centrifuged to collect the peptides at 12,000 rpm (13,780 *g*) until the ultrafiltration membrane included no surplus liquid.

iTRAQ Reagent 8-plex Multiplex kits (one unit (one vial) of reagent labels 100 µg of protein digest) were obtained from Applied Biosystems (Foster City, CA, USA) and used to label each independent biological replicate. Each iTRAQ reagent was diluted in 150 μL of isopropanol before 100 μg of peptide was added. Two replicates were labeled with 8-plex iTRAQ reagents as follows: 113, NS Stage 1; 114, NS Stage 2; 115, NS Stage 3; 116, NS Stage 4; 117, PS Stage 1; 118, PS Stage 2; 119, PS Stage 3; and 121, PS Stage 4. Another was labeled as follows: 117, NS Stage 1; 118, NS Stage 2; 119, NS Stage 3; 121, NS Stage 4; 113, PS Stage 1; 114, PS Stage 2; 115, PS Stage 3; and 116, PS Stage 4. After the samples were labeled for 1 h, 100 μL of ultra-pure high performance liquid chromatography (HPLG)-grade water, which was produced using a Barnstead Millipore water purification system (Billerica, MA, USA), was added to terminate the labeling. The samples were combined into one tube for each replicate and resolved into 50 fractions using a 5-μm particle Ultremex SCX column (Phenomenex, Torrance, CA, USA), and then they were desalted using a Gemini-NX C18 column (4.6 mm × 250 mm, Phenomenex). All the eluted fractions were freeze-dried under a vacuum. The fractions of each replicate were then resuspended with 30 μL of mobile phase A (2% acetonitrile (ACN), 0.1% formic acid (FA)) and divided into 15 groups on the basis of their peak intensities and then centrifuged at 12,000 rpm (13,780× *g*) for 10 min prior to LC-MS/MS analysis.

### 4.3. LC-MS/MS Analysis

The LC-MS/MS analysis was performed as previously described [24,64]. A splitless nanoACQuity (Waters, Milford, MA, USA) system was used for analytical separation together with a Triple TOF (SCIEX, Concord, ON, Canada). A pre-column packed with Symmetry C18 (5 μm, 180 μm × 20 mm, Waters) and analytical columns packed with BEH130 C18 (1.7 μm, 100 μm × 100 mm, Waters) were used with mobile phases A (0.1% formic acid in water) and B (0.1% formic acid in 84% ACN). Solvents, water, acetonitrile and formic acid were obtained from Thermo Fisher Scientific (Waltham, MA, USA). An 8 μL sample was loaded, and trapping and desalting were performed at 2 μL/min for 15 min with 99% mobile phase A. The samples were separated at a flow rate of 0.3 μL/min according to the following linear gradient: 0–5% mobile phase B for 5 min; 5–35% mobile phase B for 5 to 35 min; 35–60% mobile phase B for 35 min to 40 min; 60–80% mobile phase B for 40 to 42 min; 80% mobile phase B for 2 min; 80–5% mobile phase B for 44 to 45 min; and equilibration for 10 min.

The AB SCIEX Triple TOF 5600 System (Concord, MA, USA) was used to analyze the samples. Data were acquired using an ion spray voltage of 2.5 kV, curtain gas at 30 pounds per square inch (PSI), nebulizer gas at 15 PSI, and an interface heater temperature of 150 °C. The MS was operated at an resolving power (RP) of greater than or equal to 30,000 FWHM for the TOFMS scans (Agilent Technologies, Santa Clara, CA, USA). The survey scans were acquired at 250 ms, and as many as 30 product ion scans were collected when a threshold of 120 counts per second (counts/s) was exceeded, with a 2+ to 5+ charge-state for information-dependent data acquisition (IDA). The total cycle time was fixed at 3.3 s. The Q2 transmission window was set to 100 Da for 100%. Four time bins were summed for each scan at a pulse frequency of 11 kHz by monitoring the 40-GHz multichannel TDC detector via four-anode/channel detection. A sweeping collision energy setting of 35 ± 5 eV coupled with iTRAQ-adjusted rolling collision energy was applied to all the precursor ions for collision-induced dissociation. The dynamic exclusion was set to 1/2 of the peak width (18 s), and then the precursor was refreshed off the exclusion list.

### 4.4. Database Search and Quantification

The MS/MS data were used as a query in Mascot 2.3.02 (Matrix Science, Boston, MA, USA) against an integrated database, including the sequence of known full-length genes from upland cotton and protein sequences from the *G. raimondii* [27] and *G. arboretum* genomes [28]. The criteria for the database search were set as follows: the digestion enzyme was set as trypsin with one missing cleavage at an MS/MS fragment ion mass tolerance of 0.1 Da and a peptide tolerance of 0.05 Da. The carbamidomethyl of cysteine, the iTRAQ 8-plex of lysine, and the N-terminal amino group of peptides were set as fixed modifications. Simultaneously, the oxidation of methionine and the iTRAQ 8-plex of tyrosine were set as variable modifications. The peptide charge was set as Mr and the monoisotopic mass was selected. The false discovery rate (FDR), which is the result of false positive matches divided by the total number of matches, accounted for less than 1.5% in the final results. Peptides that scored as significant (≥20) at a 99% confidence interval were used during the protein identification, and each confident protein included at least one unique peptide. For protein quantification, a median was introduced as the protein ratio type to demonstrate the reproducibility of the replicates, and then the ratios for each protein were normalized using log_2_. Here, a method described by Cox [65] was used to calculate the significant changes in protein abundance. In brief, the mean and SD from the log_2_ ratios of 7388 quantified proteins was first calculated. Then, the 95% confidence (*z*-score = 1.96) was used to select the proteins whose distribution was removed from the primary distribution. Thus, the confidence interval was 1.01801367 (mean ratio of 7388 proteins) + 1.96 × 0.0820695718 (SD), corresponding to a protein ratio of 1.17887003. Finally, the protein ratios outside this range were defined as being significantly different at a *p*-value < 0.05. Therefore, the fold change (FC) cut-off for determining over- (FC > 1.2) or down-accumulated (FC < 1.2) ranges was set at 1.2 times with a *p*-value < 0.05 for at least two replicates.

### 4.5. Bioinformatics Analysis of Proteins

Based on the information obtained from the protein search, the Blast2GO software program (Available on line: http://www.geneontology.org) was used to obtain the functional classification of the proteins based on Gene Ontology (GO) terms, and the Clusters of Orthologous Groups (COG) (Available on line: http://www.ncbi.nlm.nih.gov/COG/) system software program was used to determine the protein distribution of each functional group [29]. Furthermore, a GO enrichment analysis was performed on the basis of the DAPs, and the BiNGO tool was used to perform a visualization analysis of the GO enrichment [66]. For the cluster analysis, a *K*-Means clustering analysis with a Euclidean distance metric and a Calculate *K*-Means clustering method was used. The BLASTX software program was used to compare our proteins with the *Arabidopsis* protein database using The Arabidopsis Information Resource (TAIR) online (Available on line: http://www.arabidopsis.org/).

### 4.6. RNA Isolation and Quantitative Real-Time PCR (qPCR)

The total RNA was isolated using an RNAprep Pure Plant Kit (Polysaccharides & Polyphenolics-rich) (TIANGEN, Cat.＃DP441, Beijing, China) from cotton leaves that were stored at −80 °C according to the manufacturer’s protocol. Extra RNA was stored at −80 °C, and the concentration was determined using a UV-visible spectrophotometer mini-1240 (SHMADZU, Kyoto, Japan). The corresponding volume of 1 μg of RNA was quantified according to the concentration. Then, 1 μg of RNA was reverse-transcribed into cDNA using the PrimeScript^™^ RT reagent kit with a gDNA Eraser (Perfect Real Time) (TaKaRa, Cat.#RR037A, Kusatsu, Japan). qPCR was then performed on an ABI 7500 real-time PCR system (Applied Biosystems) equipped with ULtraSYBR Mixture (Low ROX) (CWBIO, Cat. CW2601M, Beijing, China), and the reaction system is as follows: 2× ULtraSYBR Mixture (2 μL), PCR primers (0.4 μL), cDNA (2 μL), and ddH_2_O (7.2 μL). Three independent replicates were performed for each sample. The data were processed using the 2^−ΔΔ*C*t^ method, and *GhActin* and NS Stage 1 were used as internal controls. All of the primer pairs (Table S9) used for qPCR were designed using Oligo 7 [67].

### 4.7. Chlorophyll, MDA, Plant Hormone and Hydrogen Peroxide Content Determination

The chlorophyll and MDA levels are often used as markers for the progression of senescence because the levels of both change significantly during this process [20]. A UV-visible mini-1240 spectrophotometer (Shmadzu, Japan) was used to perform the experiment using the method described by Lichtenthaler [68]. For each sample, 0.2 g samples of fresh cotton leaves in triplicate were cut into pieces and the midrib was removed. Acetone (10 mL) was added to a mortar and the sample tissue was ground before it was filtered into a brown volumetric flask (25 mL) and then diluted with 80% acetone to 25 mL. The absorbance values of the extract were measured at 645 nm and 663 nm. We calculated the chlorophyll concentrations according to the following formula: C_a+b_ = 20.3 D_645_ + 8.04 D_663_ [69].

The MDA was detected according to the method described by Saher [70]. For each sample, 2 mL of 10% trichloroacetic acid (TCA) was added to 1 g of fresh cotton leaves in triplicate, and the samples were homogenized by milling with quartz sand. Another 8 mL of 10% TCA was added before the homogenate was transferred to a 15 mL centrifuge tube. After centrifugation for 10 min at 4000 rpm (1530× *g*), 3 mL of supernatant was transferred to a new tube. After 3 mL of 0.6% thiobarbituric acid (TBA) was added, the samples were incubated in boiling water for 15 min. Each sample was then cooled to ambient temperature and centrifuged for 10 min at 4000 rpm (1530× *g*). The absorbance values were measured at 532 nm, 600 nm and 450 nm. We measured the MDA content using the following formula: Concentration of MDA (μmol/L) = 6.45 × (OD_532_ − OD_600_) − 0.56 × OD_450_; Content of MDA (μmol/g) = Concentration of MDA (μmol/L) × Volume of extract solution (L)/Tissue weight (g).

The plant hormones were detected according to Li’s procedure [71] and the appropriate modifications were made. After 0.2 g of fresh cotton leaf samples were weighed in triplicate for each sample, 2 mL of extract (n-propanol/water/hydrochloric acid) was added at a ratio of 200:100:0.2 (*v*/*v*/*v*) and then shaken for 30 min in a light-shielded ice bath. A 1 mL volume of dichloromethane was added before another 30-min shaking session under light shielding in the ice bath. After centrifugation for 10 min at 4500 rpm (1940× *g*), 1 mL of subnatant was transferred to a new tube, and another 1 mL of dichloromethane was added to the residue of the original tube, and the above steps were then repeated. A 2 mL volume of subnatant was blow-dried using nitrogen blowing before 1 mL of 80% methyl alcohol was added and the mixture vortexed for 5 min. The CNWBOND C18 column was equilibrated with 10 mL of methanol and 10 mL of 80% methanol solution, and then the supernatant was eluted into a brown vial. All the eluted samples were dried under a vacuum at 35 °C. The samples of each replicate were then resuspended with 200 μL of methanol and 0.05% methanol solution at a ratio of 1:1 (*v*/*v*). Finally, all the samples were centrifuged for 5 min at 12,000 rpm (13,780× *g*) and then the supernatant was transferred to a new tube to perform High performance liquid chromatography electrospray ionization mass (HPLC-ESI-MS) analysis.

Hydrogen peroxide was detected using the following process. First, a standard curve was prepared before the hydrogen peroxide in the cotton leaves was determined. The reagents listed in Table S10 were added to different tubes, and all of them were placed in the dark for an hour. The absorbance values at 390 nm were then measured to draw the standard curve. Second, the concentrations in the cotton leaves were determined according to the following procedures: After 0.2 g of fresh cotton leaves were weighed in triplicate for each sample, 5 mL of 0.1% TCA was added, and the samples were centrifuged for 20 min at 10,000× *g* (10,223 rpm). Phosphate buffer (0.01 mol/L PBS, 1 mL) and potassium iodide solution (1 mol/L, 2 mL) were sequentially added to the supernatant (1 mL), and then the absorbance values were measured at 390 nm. The hydrogen peroxide content, which was defined as value 1, was calculated according to a standard curve before the ultimate concentration was determined using the formula. Finally, we measured the ultimate H_2_O_2_ concentration using the following formula: Concentration of H_2_O_2_ (μmol/g FW) = (Value 1 × Volume of TCA)/(Volume of supernatant × FW).

## 5. Conclusions

Here, 443 DAPs among 7,388 high-confidence proteins were detected using 8-plex iTRAQ tests between NS and PS at four leaf stages. All of the DAPs were grouped into 9 clusters based on their accumulation profiles, including 3 over-accumulated clusters in PS. GO enrichment analysis showed that DAPs associated with abiotic stress, and especially those responding to cold temperatures, are differentially accumulated during the four stages. Fifty-eight proteins were significantly related to abiotic stress, hormone signaling and leaf senescence, and 26 CRPs were significantly differentially accumulated. A decline in temperatures before the onset of aging is believed to be the primary inducer. H_2_O_2_ and JA were found to be increased in PS. Thus, it is firmly believed that the accumulation of H_2_O_2_ and the JA regulatory pathway are two factors that shape cold-induced cotton leaf senescence.

## Supplementary Materials

Supplementary materials can be found at www.mdpi.com/1422-0067/18/9/1984/s1.
