# Supplementary figures and images for: iTRAQ-Based Quantitative Proteomic Analysis Reveals Cold Responsive Proteins Involved in Leaf Senescence in Upland Cotton (Gossypium hirsutum L.)
Source: Int J Mol Sci. 2017 Sep 16;18(9):1984. doi: 10.3390/ijms18091984 (PMC5618633; doi:10.3390/ijms18091984)

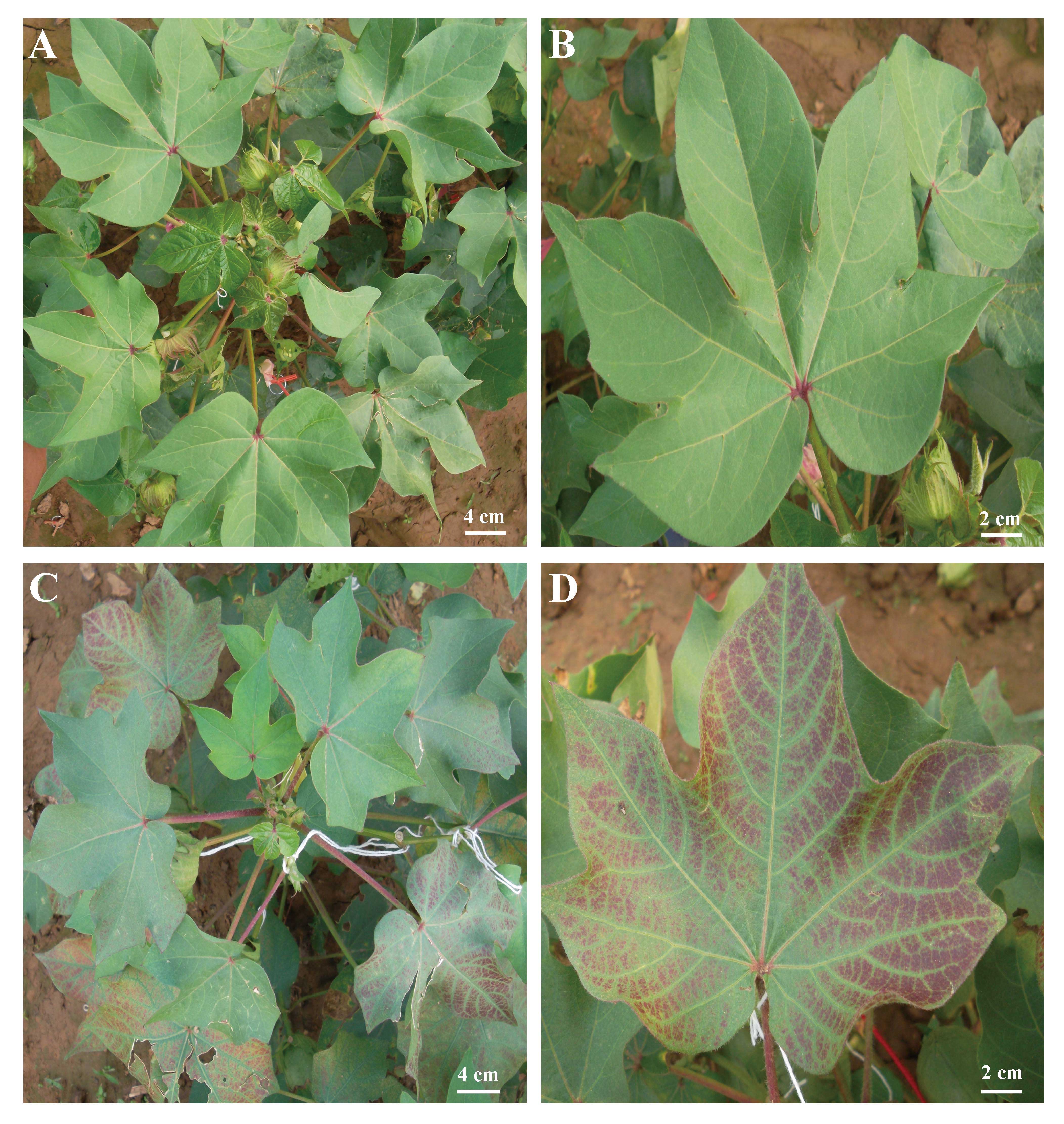

Supplement: Supplementary file 1 [file ijms-18-01984-s001.zip › ijms-223259-for final-supplementary/Fig.S1.jpg]

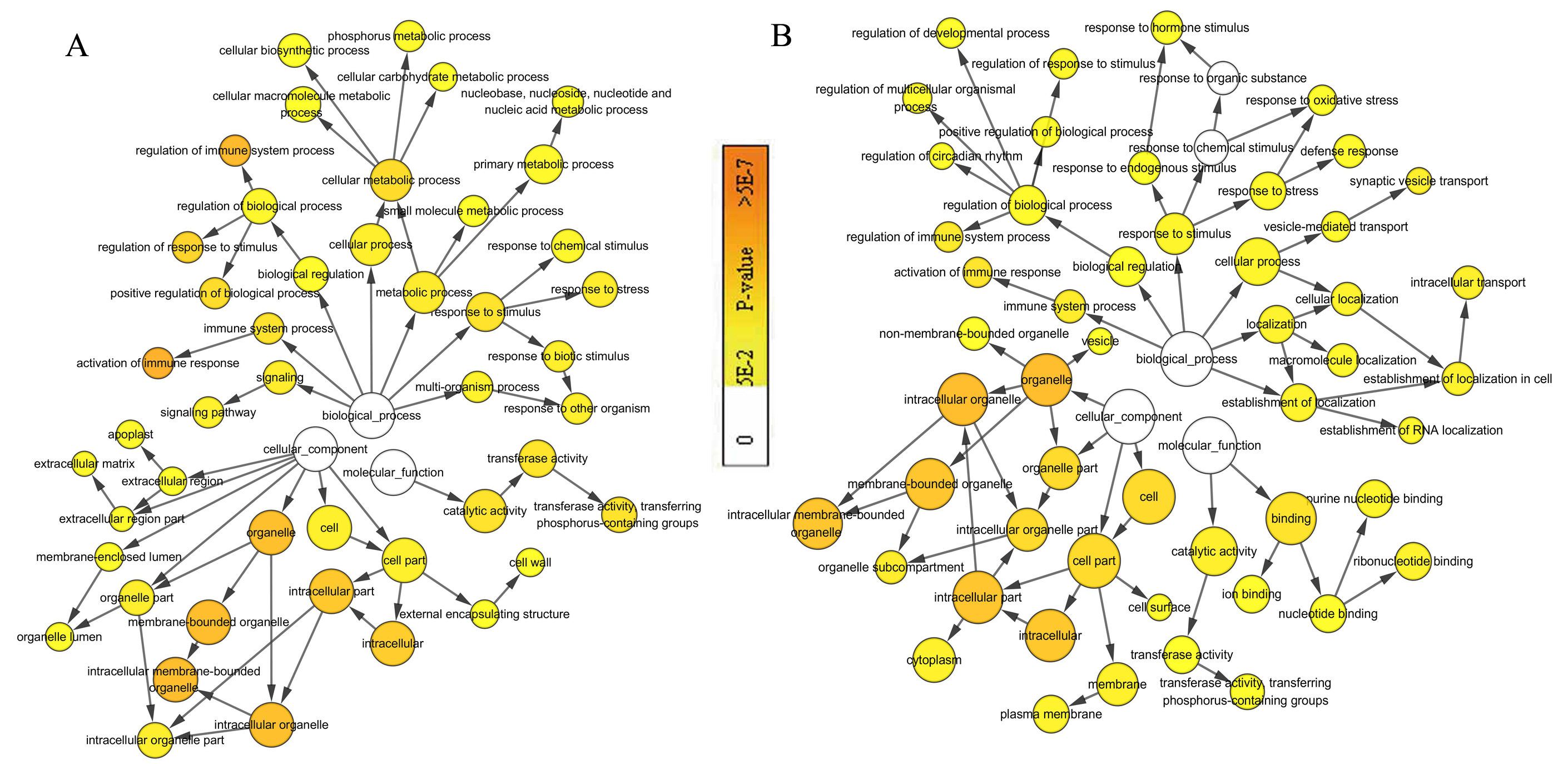

Supplement: Supplementary file 1 [file ijms-18-01984-s001.zip › ijms-223259-for final-supplementary/Fig.S2.jpg]
